# Surface‐engineered bacteria in drug development

**DOI:** 10.1111/1751-7915.70033

**Published:** 2024-10-15

**Authors:** Charles Dahlsson Leitao, Stefan Ståhl, John Löfblom

**Affiliations:** ^1^ Department of Protein Science KTH Royal Institute of Technology Stockholm Sweden

## Abstract

Bacterial surface display in combination with fluorescence‐activated cell sorting is a versatile and robust system and an interesting alternative approach to phage display for the generation of therapeutic affinity proteins. The system enables real‐time monitoring and sorting of cell populations, which presents unique possibilities for drug development. It has been used to develop several affibody molecules currently being evaluated preclinically for the treatment and diagnosis of, for example, cancer and neurodegenerative diseases. Additionally, it can be implemented in other areas of drug design, such as for mapping epitopes and evolving enzyme specificities.

## Graphical abstract

Bacterial surface display in combination with fluorescence‐activated cell sorting is a versatile and robust system for the generation of therapeutic affinity proteins. It has been used to develop affibody molecules for the treatment and diagnosis of, for example, cancer and neurodegenerative diseases.

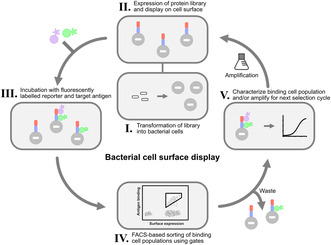

## INTRODUCTION

Phage display has been the predominant technology for the development of affinity proteins with novel binding capabilities ever since its conception (Smith, 1985) and has been the source of numerous monoclonal antibodies approved for various medical conditions (Alfaleh et al., 2020). While phage display is undoubtedly an invaluable technology for the generation of novel affinity proteins, particularly monoclonal antibodies, other display technologies might prove situationally more useful for drug development by providing a unique approach with distinct advantages. This review will delineate how fluorescence‐activated cell sorting (FACS) used in combination with bacterial cell display can complement phage display in the development of affinity proteins for medical applications, with an emphasis on the generation of affibody molecules for cancer therapy. Affibody molecules are small alternative scaffold proteins that can be robustly generated with high affinity towards a wide range of target proteins using bacterial cell display, and examples will be given of such affibody‐based drugs currently in preclinical development. Both *Staphylococcus carnosus* and *Escherichia coli* will be described, detailing some of the work that has been done to optimize these systems with flexible solutions to drug development that are difficult to implement in a phage‐based system, thus offering a versatile toolbox for the development of protein‐based drugs.

## BACTERIAL SURFACE DISPLAY

**FIGURE 1:**
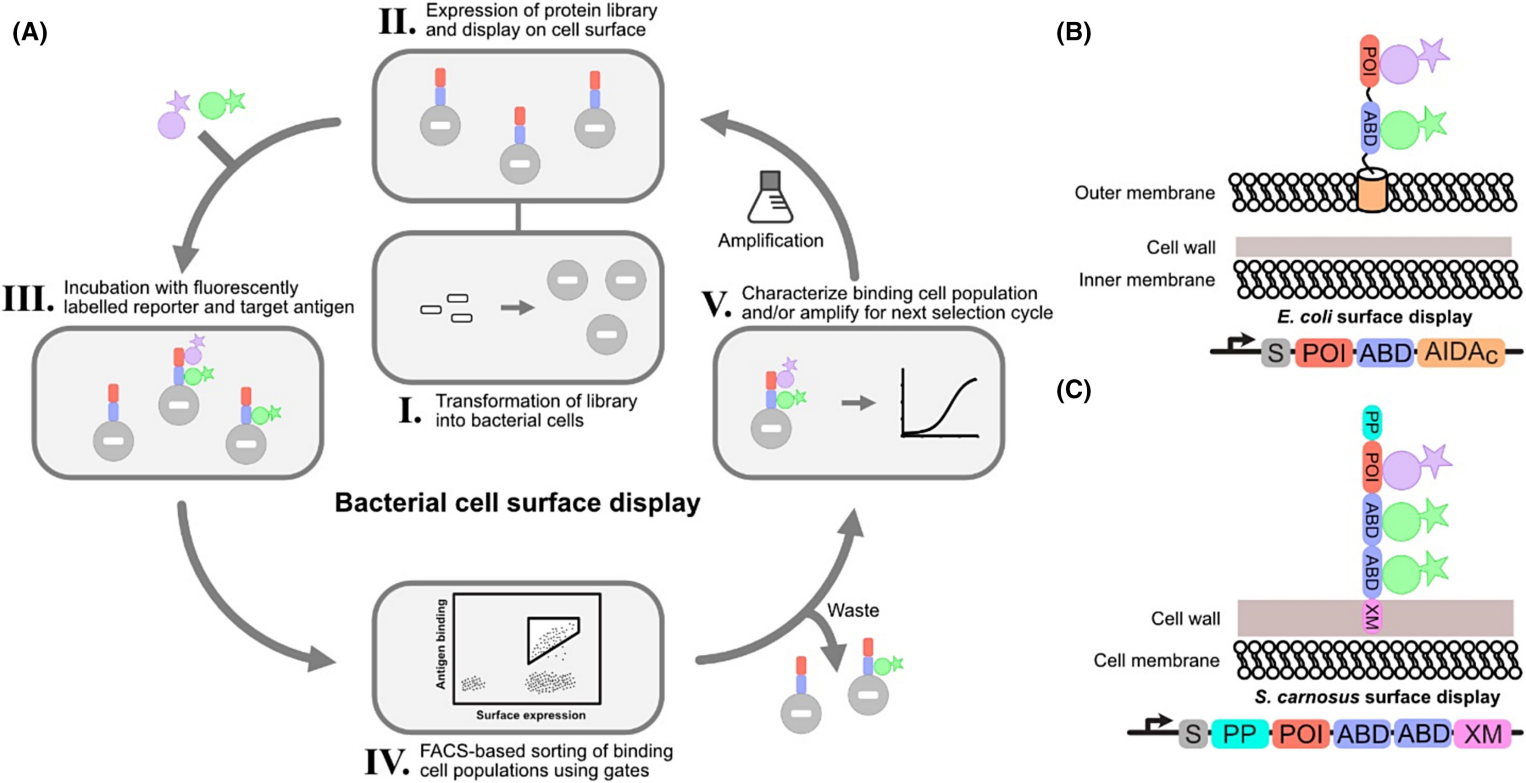
**(**A) Schematic overview of bacterial cell surface display methodology. I. A DNA library coding for millions or billions of different protein variants is used to transform bacterial cells, typically by heat shock or electroporation methods. II. Cells express the protein library, often fused to a normalization tag such as an albumin‐binding domain (ABD), and display it on the surface through different cellular mechanisms. III. The cells are incubated with fluorescently labelled target antigen and reporter (for example, human serum albumin). Here, the stringency of the selection round can be adjusted by changing the number of washing steps or dissociation times. IV. The cells are sorted through a flow cytometer to be discarded or kept depending on the desired level of fluorescent signal determined through gating. V. The entire binding cell population can be amplified for additional selection rounds or single clones can be analysed by sequencing and by direct binding analysis using flow cytometry. (B) Mechanisms by which the protein library (POI) can be displayed on the cell surface. For display on the Gram‐negative *E. coli*, a method using the autotransporter secretion pathway of AIDA‐I can be used to express, secrete and anchor the protein of interest on the cell surface. (C) For display on Gram‐positive *S. carnosus*, a cell‐wall anchoring sequence (XM) is used to anchor the protein library on the cell wall following secretion, directed by the signal peptide (S), through the cell membrane.

In a cell‐based system, protein libraries are anchored and displayed on the cell surface, thereby creating a physical link between the phenotype and the encapsulated genotype, allowing for the recovery of functional sequences after selections. Moreover, due to the markedly larger size of bacterial cells compared to bacteriophages, functional cell populations can be sorted from non‐functional cell populations based on fluorescent signals in real‐time using FACS (Löfblom, 2011). Furthermore, an advantage with the use of a fluorescent‐based system is that multiple fluorescent signals can be interrogated simultaneously, which enables more than one functionality, such as for bispecific binders, to be assessed during the selection procedure (Nilvebrant et al., 2014). While it is possible to fuse a protein library to the coat protein pVIII of bacteriophages to display many copies on the surface, this typically results in low‐affinity binders because of strong avidity effects during biopanning; thus, protein libraries are often fused to pIII for monovalent display. However, for FACS‐based cell display, multivalent expression of protein libraries turns out to be an advantage as it allows for the quantification of relative affinity among binding clones (Löfblom et al., 2005). This is because of the inclusion of reporter domains, such as, in our systems, an albumin‐binding domain, that are used for normalization of surface expression by the addition of a fluorescently labelled reporter, in this case human serum albumin (Jonsson et al., 2008). Additionally, the fluorescently labelled reporter protein, binding the normalization tag, and the target proteins are free in solution and not immobilized to physical support, thus eliminating the risk for avidity‐driven binding events. The possibility to discriminate affinities in real‐time and select for a desired range in binding affinity, especially during affinity maturation campaigns, is valuable for the development of effective and specialized treatment modalities with desired characteristics. A schematic overview of FACS‐based bacterial display selections is shown in Figure 1A.

Bacterial display is not without limitations, however. For highly complex proteins or proteins dependent on post‐translational modifications for an accurate representation of function, FACS‐based selection systems are still viable but are typically limited to yeast or mammalian cell display technologies. Due to limitations of the rate at which cells are processed through a flow cytometer, a reduction in library complexity is often necessary to allow for oversampling during FACS‐based selections. Magnetic‐assisted cell sorting (MACS) is a methodology utilizing paramagnetic beads with immobilized target antigen to capture an initially broad binding cell population due to avidity‐driven binding and thereby retain a smaller but significant portion of the initial library (Parks et al., 2024).

The characteristics of the bacterium used for the surface display of protein libraries will confer the utility and limitations of the system. The Gram‐positive *S. carnosus* has been evaluated for cell surface display of recombinant proteins because of several interesting features (Dahlsson Leitao, Mestre Borras, Jonsson, et al., 2023; Löfblom et al., 2017; Samuelson et al., 1995). It has the advantage of lacking a second membrane, which facilitates translocation of the protein library to the cell surface. Furthermore, cells have higher tolerance to mechanical stress due to the thick peptidoglycan cell wall and are thus better adapted to endure the harsh conditions induced by the flow cytometer during cell sorting, resulting in higher cell viability (Löfblom, 2011). The transformation efficiency for *S. carnosus* was initially low, mostly due to the thicker cell wall, limiting the size of practical protein libraries. However, due to improvements to the transformation protocol, including knocking out host restriction enzymes by heat treatment, the transformation efficiency increased 10,000‐fold (Löfblom et al., 2007, 2023). This allowed for high‐complexity libraries of the size 10^9^ to be constructed, opening the possibility for both naïve and maturation libraries to be used with *S. carnosus* display. A schematic overview of how a protein library can be displayed on the surface of *S. carnosus* is shown in Figure 1C. The expression vector contains a secretion signal sequence (S) and a propeptide (PP) derived from a gene found in *Staphylococcus hyicus* expressing a membrane‐bound lipase. The protein library is inserted using cloning sites downstream of the PP. Two high‐affinity ABDs are included downstream of the library for normalization of surface expression. A cell‐wall anchoring sequence (XM) derived from staphylococcal protein A is included at the C‐terminus and is recognized by endogenous sortase, which cross‐links the displayed protein construct to the peptidoglycan cell wall, thus anchoring it to the cell surface (Löfblom et al., 2017).

The most extensively studied bacterial host for displaying peptide and protein libraries on the surface is the Gram‐negative *E. coli*, owing to its many attractive features such as high transformation efficiency, fast doubling time, robustness, high expression levels, and ease of use (Daugherty, 2007), with optimized protocols for library construction and selection procedures readily available (Dahlsson Leitao, Hjelm, Ståhl, et al., 2023; Ståhl, Hjelm, et al., 2023). Additionally, because of its ubiquitous use in research, there is a trove of information concerning, for example, optimal cloning procedures, recombinant protein expression, and secretion systems. The high transformation efficiency of *E. coli* allows for practical protein libraries comprising 10^11^ variants, which is comparable to what can be achieved with phage display (Parks, 2024).

Display of the protein library on the surface can be achieved by utilizing autotransporter secretion pathways, of which there are several (Jose & Meyer, 2007). The type V autotransporter Adhesion Involved in Diffuse Adherence (AIDA‐I) has been used to efficiently express, secrete, and anchor protein libraries on the surface of *E. coli* (Fan et al., 2016; Leo et al., 2012). The AIDA‐I autotransporter pathway involves a signal peptide sequence directing the unfolded passenger sequence, or in this case the protein library, for translocation across the inner membrane. The β‐barrel domain called AIDAc folds in the outer membrane with the assistance of chaperones and shuttles the passenger sequence across, where it becomes anchored to the outer membrane. A schematic example of displaying protein libraries on the surface of *E. coli* using the AIDA‐I autotransporter for FACS‐based selections and the corresponding expression vector are shown in Figure 1B. Optimization of the AIDA‐I expression vector was performed to reduce the size of the passenger sequence by introducing a shorter linker and a single high‐affinity ABD. Based on this new vector, a large naïve combinatorial affibody library was constructed and evaluated on the surface of *E. coli* by mounting a selection campaign against several targets of varying size, including HER2, HER3, IL3RA, CD69 and DGCR2 (Andersson et al., 2019). For all targets except IL3RA, clones with binding affinities in the nanomolar range were obtained and verified using flow cytometry and biosensor assays, exemplifying the robustness of the *E. coli* display system for affinity protein discovery. Further improvements to the AIDA‐I‐based *E. coli* display system have been made, regarding both the expression vector architecture and the protocols for the selection of affibody molecules (Parks et al., 2024).

## DRUG DEVELOPMENT USING BACTERIAL DISPLAY

**FIGURE 2:**
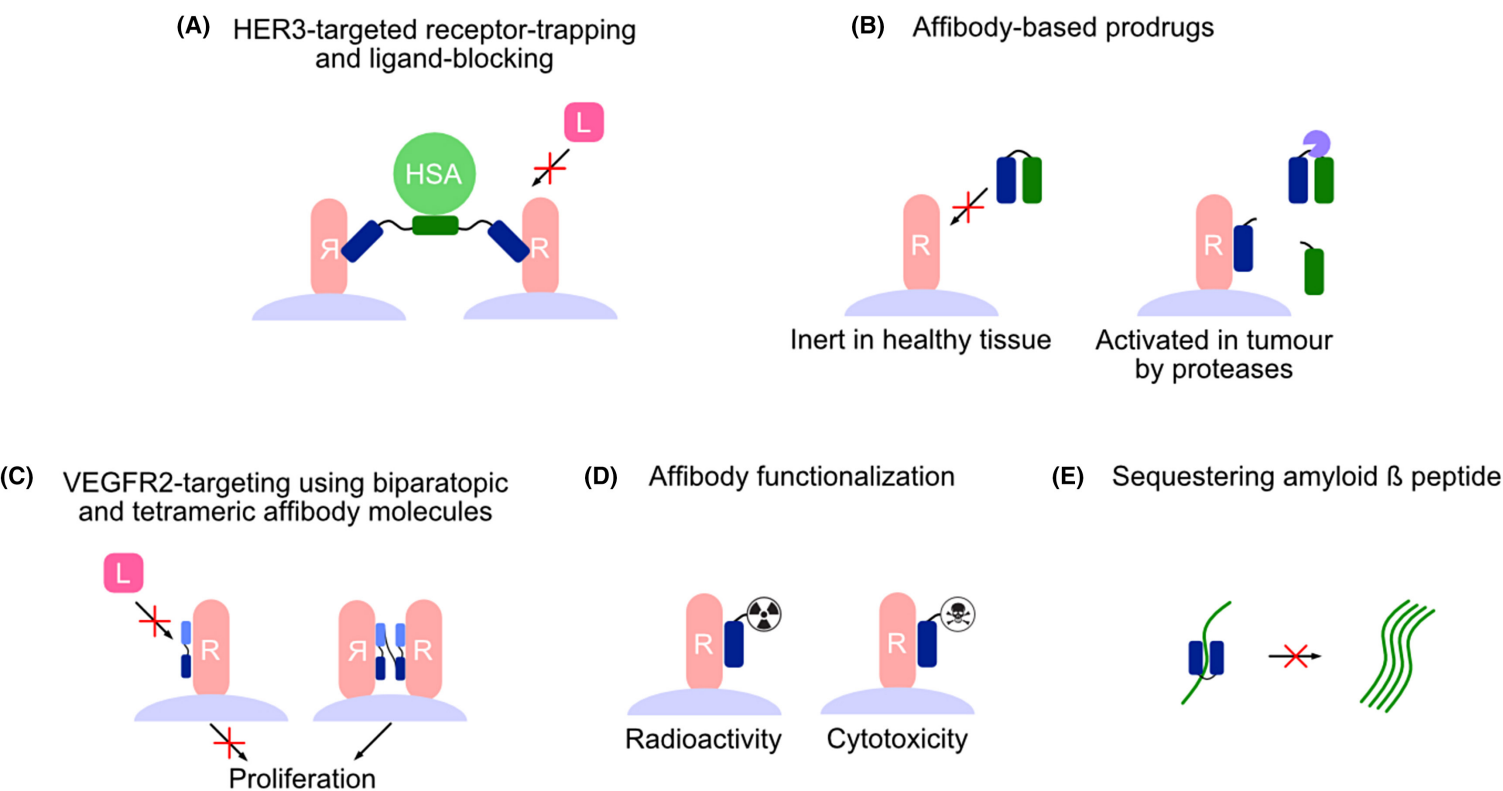
Illustration of drug strategies that have been explored using affibody molecules generated from bacterial cell display. (A) A bivalent HER3‐targeting affibody molecule comprised of two identical HER3‐binding domains that bind an epitope overlapping with that of the natural ligand heregulin and an albumin‐binding domain that prolongs the circulatory half‐life by association with human serum albumin (HSA). (B) An EGFR‐binding affibody molecule is fused by a protease substrate linker sequence to an anti‐idiotypic affibody masking domain with affinity for its binding surface, allowing the drug to be conditionally activated in protease‐rich tumour environments while remaining inert in circulation and healthy tissues. (C) VEGFR2‐targeting affibody molecules that either act as an inhibitor for the ligand VEGF when formatted as a biparatopic molecule but promote proliferation and angiogenesis when formatted as a tetrameric molecule by acting as an artificial ligand. (D) Affibody molecules can be functionalized site‐specifically using unique cysteine residues with, for example, cytotoxic small molecular drugs for increasing drug potency or radioactive compounds for diagnostic radioimaging applications. (E) Affibody‐based sequestrin molecules with a unique conformation that binds to monomeric amyloid ß peptides by enveloping them with high affinity, thus preventing the formation of large aggregates.

Bacterial surface display fills an important niche in the expansive landscape of therapeutic affinity protein development, being particularly useful and versatile for less complex protein scaffolds. Thus, it has been and is continuously being used for the development of therapeutic affibody molecules. A selection of affibody molecules generated from bacterial cell display that are currently in preclinical development will be covered in this review. Information related to their selection is presented in Table 1, and illustrations related to the drug strategies that have been employed using them are presented in Figure 2. Additionally, affibody‐based candidate pharmaceuticals are currently being evaluated in late‐stage clinical trials (Klint et al., 2023), but will not be covered here. Affibody molecules are small (58 amino acids, 6–7 kDa) helical bundle proteins derived from the B‐domain of staphylococcal protein A (Ståhl et al., 2017). The scaffold is highly resilient to harsh conditions and can refold to its native functional state within 3 μs following heat‐ and chemical‐induced denaturation (Arora et al., 2004), which presents unique possibilities for both drug design and production. For example, permitting temperatures up to 95°C enables rapid radiolabelling of macrocyclic metal chelators for diagnostic radioimaging applications (Altai et al., 2013; Strand et al., 2013), and it allows for the removal of heat‐sensitive host proteins during production. Furthermore, many affibody molecules remain unaffected in a broad range (2.8–11.0) of pH values (Ekblad et al., 2008; Engfeldt et al., 2007) and in the presence of lipophilic inorganic solvents, making purification highly flexible and efficient (Heskamp et al., 2012).

**TABLE 1:** Examples of target antigens that have been used for the bacterial cell surface display selections of affibody molecules currently in preclinical development.

Target antigen	Target size (kDa)	Highest affinity	Cross‐reactive murine	Selection method	Library type
Amyloid ß peptide (Aß)	4.5	0.3 nM	Yes	*Staphylococcus carnosus* display	Maturation
HER3	69 (ECD)	21 pM	Yes	*S. carnosus* display	Maturation
VEGFR2	83 (ECD)	0.2 nM	Yes	*S. carnosus* display	Maturation
Z_EGFR:2377_	7	568 nM	N/A	*S. carnosus* display	Naïve
CD69	17 (ECD)	30 nM	Yes	*Escherichia coli* display	Naïve

Affibody libraries for bacterial display selections are constructed by randomization of 13–15 surface exposed residues on helices one and two during DNA library synthesis using trinucleotide codon mixtures of predefined composition, resulting in highly controlled and functional diversification (Arunachalam et al., 2012). Affibody libraries typically exclude prolines and glycines because of their propensity to break helices, and cysteine to enable the functionalization of affibody molecules post‐selection with the inclusion of solitary cysteine residues at specific positions (Ståhl, Lindberg, et al., 2023). Depending on the intended application, these unique cysteine residues are a crucial aspect of the drug design, as they are typically used to, for example, introduce metal chelators for labelling of radioactive compounds or conjugate chemotherapeutic drugs in the development of affibody‐drug conjugates (Figure 2D). Perhaps the most prominent feature of affibody molecules is the possibility of developing binders that are specific and with extraordinarily high affinity despite their minuscule size and relatively flat binding surface. The small size is especially desirable for diagnostic radioimaging applications as it translates to rapid tissue extravasation and diffusion, resulting in efficient tumour localization, and rapid excretion from the blood by glomerular filtration, resulting in low background and high imaging contrasts (Tolmachev & Orlova, 2020). To demonstrate the difference in potential utility for diagnostic radioimaging of human epidermal growth factor receptor 3 (HER3), a gallium‐68 labelled HER3‐binding affibody molecule (Z_HER3:08698_) was compared to zirconium‐89 labelled HER3‐binding antibody Seribantumab and its corresponding F(ab’)_2_‐fragment (Rinne, Leitao, et al., 2021). The affibody demonstrated superior imaging properties for visualization of HER3‐expressing tumours in mice already at 3 h post‐injection compared to both the F(ab’)_2_‐fragment and the full‐length antibody at 48 and 96 h, respectively, corresponding to their most favourable imaging time‐points.

The HER3‐binding affibody molecule Z_HER3:08698_ was developed from an affinity maturation library using *S. carnosus* display (Kronqvist et al., 2011; Malm et al., 2013) and has a remarkably high monovalent affinity of 21 pM, which is the highest attained for affibody molecules. Due to the small size of affibody molecules, minute changes to the composition can have a dramatic effect on its behaviour in circulation while its binding affinity and specificity remain unaffected. For this reason, a large effort has been made to optimize the design of affibody‐based imaging tracers to improve tumour targeting properties and biodistribution (Rinne, Orlova, & Tolmachev, 2021). As an example, the HER3‐binding affibody molecule used in the above‐mentioned comparative study was the culminative product of many years of optimization (Dahlsson Leitao et al., 2019; Rinne, Leitao, et al., 2019; Rinne et al., 2019; Rinne et al., 2020; Rinne, Xu, et al., 2020; Rosestedt et al., 2019). Additionally, since the binding site of Z_HER3:08698_ overlaps with that of the natural ligand heregulin, it also provides an avenue for therapeutic intervention by inhibiting ligand‐induced activation of the HER3 receptor. However, rapid excretion due to the small size, which for diagnostic radioimaging is an advantage, becomes a problem when high bioavailability is necessary to ensure continuous exposure to a drug. This can be solved by genetically fusing an albumin‐binding domain to the affibody molecule, which will increase the hydrodynamic radius by the association to abundant human serum albumin in blood (Figure 2A) (Hoogenboezem & Duvall, 2018; Jonsson et al., 2008). This is possible because of the high modularity of affibody molecules, whereby fused affibody domains can act independently with typically only a small cost to affinity and stability. It was shown that the valency of Z_HER3:08698_ and the orientation with respect to an ABD heavily influenced targeting properties and biodistribution, demonstrating that careful design of therapeutic affinity proteins is essential for proper function (Altai et al., 2018). Moreover, the most promising affibody variants were comparable to a clinically evaluated HER3‐targeting antibody in terms of tumour growth inhibition and prolonged survival of mice harbouring HER3‐expressing pancreatic cancer xenografts (Dahlsson Leitao et al., 2020). The concept of therapeutic HER3‐targeting affibody molecules was further developed by site‐specifically conjugating the affibody variant exhibiting the highest therapeutic potential with the chemotherapeutic drug DM1 and evaluating its targeting properties and biodistribution in vivo (Rinne et al., 2022). Studies looking at the preclinical therapeutic efficacy of DM1‐conjugated HER3‐targeting affibody molecules in tumour‐bearing mice have been performed, and the results look promising (Zhang et al., 2024).

Attaching potent chemotherapeutic drugs or other toxins to affinity proteins is an attractive strategy to effectively eliminate cancer cells with 11 antibody‐drug conjugates (ADCs) currently approved by the FDA for treatment of various cancers (Gogia et al., 2023). What most of these ADCs have in common, however, is that they target antigens that are either specifically expressed in a particular cancer or highly overexpressed compared to healthy tissues. Targeting cancer‐associated antigens that are ubiquitously expressed will in many cases result in severe and potentially life‐threatening side effects. Human epidermal growth factor receptor (EGFR) is a cancer biomarker that is overexpressed in several cancers (Thomas & Weihua, 2019), for example, non‐small cell lung cancer, but also exhibits relatively high expression in other healthy tissues, such as skin and liver (Lacouture et al., 2018). Designing drugs that become conditionally active in the tumour microenvironment while remaining inert in circulation might resolve some of the limitations associated with drug conjugates. For this purpose, an anti‐idiotypic masking domain (Z_B05_) with an affinity for the binding surface of an EGFR‐targeting affibody molecule (Z_EGFR_) was generated using *S. carnosus* display (Mestre Borras et al., 2023). A prodrug was constructed by fusing the masking domain Z_B05_ to Z_EGFR_ using a linker containing a protease substrate sequence (Figure 2B). The affibody prodrug concept has been evaluated in preclinical mouse models using H292 lung cancer xenografts (Dahlsson Leitao, Mestre Borras, Xu, et al., 2023). Conditional activation of the prodrug in the xenografted tumour, mediated by cancer‐associated proteases catalysing the removal of the masking domain, could be observed. The uptake of the prodrug in the liver was 6‐fold lower compared to a positive binding control, suggesting that the prodrug remains mostly intact and inert in circulation.

Related to the development of prodrugs activated by tumour‐associated proteases, bacterial surface display has been used for the discovery of substrate sequences that are more efficiently cleaved by a given enzyme. An affinity protein was interconnected with an anti‐idiotypic masking domain using a linker comprising a library of enzyme substrates and displayed on the cell surface (Sandersjöö et al., 2017). Substrate sequences recognized by the enzyme would be cleaved and thus release the masking domain, allowing for FACS‐based sorting of cells with unmasked affinity proteins that can bind fluorescently labelled target antigens. Through this method, new substrate sequences for metalloprotease (MMP)‐1 and tobacco etch virus (TEV) protease that are cleaved up to eight times more efficiently than previously reported substrates were selected using *S. carnosus* display. Similar selections have been made using *E. coli* display to improve substrates recognized by other proteases (unpublished results).

Another interesting therapeutic concept derives from the development of vascular endothelial growth factor receptor 2 (VEGFR2)‐specific affibody molecules. By using an affinity maturation library and *S. carnosus* display, two antagonistic binders were selected targeting two non‐overlapping epitopes on VEGFR2 with K_D_ values of 5.0 and 10.9 nM, respectively. They were formatted into a biparatopic binder capable of simultaneous binding to both epitopes, resulting in extraordinarily slow dissociation from VEGFR2 and inhibition of VEGF‐induced activity with a K_D_ of approximately 0.2 nM against both human and murine VEGFR2 (Figure 2C) (Fleetwood et al., 2015, 2016). The authors could further demonstrate that the binder strongly inhibited VEGF‐induced proliferation and sprouting of human endothelial VEGFR2‐expressing cells. Providing effective treatments to attenuate angiogenesis has important implications for several pathologies, such as various cancers and vascular eye disorders. Moreover, by formatting the antagonistic binder into a tetrameric construct consisting of two fused biparatopic domains, it could act as an artificial VEGFR2 ligand, conveying agonistic effects on angiogenesis with applications within the field of tissue engineering (Figure 2C) (Güler et al., 2019).

Moving away from cancer therapy, a large effort has been made to develop affibody molecules for the treatment of Alzheimer's disease (AD). For this, *S. carnosus* display has been combined with uniquely structured libraries designed for the selection of so‐called sequestrins that can bind and sequester monomeric amyloid beta (Aβ) peptides (Figure 2E) (Lindberg et al., 2013, 2015). The libraries consisted of two truncated affibody molecules fused into a single‐chain dimeric format. The libraries were either symmetrical, meaning that the same residues were randomized in both truncated affibody domains, or asymmetrical, meaning that different residues were randomized in the two domains. Both libraries produced binders with similar affinities, but the most interesting candidate, Z_SYM73_, was generated from the asymmetrical library with an affinity of 340 pM and evolved to have a longer linker than initially designed. The therapeutic effect of ABD‐fused Z_SYM73_ was evaluated in vivo using an AD mouse model, with results showing alleviation of Aβ plaque burden and reduced cognitive decline (Boutajangout et al., 2019). Furthermore, Z_SYM73_‐ABD has been fused to a single‐chain variable fragment binding to the transferrin receptor acting as a shuttle across the blood–brain barrier to increase the bioavailability of the affibody molecule in the brain (Faresjö et al., 2022).

Another approach, which accentuates the versatility of bacterial display systems, focuses on engineering the specificity of tobacco‐etch virus (TEV) enzyme, rather than the substrate sequence, towards recognizing Aβ peptide (Meister et al., 2023). A rationally designed TEV enzyme library with additional mutations from error‐prone PCR was co‐transformed with a gene encoding GFP rendered inactive by the fusion to an aggregation‐prone Aβ peptide. The GFP and the Aβ peptide were interconnected by the new desired enzyme substrate. The restoration of GFP activity from the removal of the Aβ peptide through cleavage by the TEV enzyme could be observed and selected, for which several new TEV variants with catalytic activity for the new substrate sequence were produced.

Inflammation is associated with a myriad of diseases and usually appears in early progression, which presents a possibility for improving disease prevention and treatment by early detection. Cluster of differentiation 69 (CD69) is expressed on the cell surface of activated lymphocytes; in addition, other immune cell populations also express CD69 throughout the body (Cibrián & Sánchez‐Madrid, 2017; Kimura et al., 2017; Radulovic & Niess, 2015). Currently, only antibodies have been developed for high‐affinity CD69‐targeting with the limitations, discussed previously, for diagnostic imaging applications due to, among other things, slow blood clearance resulting in late imaging time‐points and poor contrasts. Using an *E. coli* display, an affibody molecule targeting human CD69 was generated with cross‐reactivity for the murine analogue and subsequently developed further using an affinity‐maturation *E. coli* library, resulting in a panel of binding variants with a range of affinities for human and murine CD69 (Persson et al., 2021). They were characterized preclinically in mice as indium‐111 radiolabelled imaging probes for the detection of CD69 as an inflammation marker on activated lymphocytes using SPECT. The most promising variant exhibited an affinity of approximately 30 nM for both human and murine CD69, high stability, and favourable biodistribution with a clear uptake in lymph nodes.

Another interesting application of bacterial cell display for drug development is the mapping of epitopes. *S. carnosus* has been used to display libraries of peptide stretches of varying lengths covering the entire protein sequence of different antigens recognized by monoclonal and polyclonal antibodies (Hjelm et al., 2010, 2012; Rockberg et al., 2008, 2010). The epitopes could be identified by analysing the regions of the antigen recognized by fluorescently labelled antibodies during library screening.

## CONCLUDING REMARKS

By examining the strengths and weaknesses of different technologies, we can learn to complement each when necessary. While phage display is a powerful method for developing affinity proteins, especially antibodies, from large naïve libraries, bacterial display is best suited for less complex protein scaffolds, such as affibody molecules, and excels at smaller, more focused libraries. Additionally, the scope for bacterial display applications extends further than the generation of affinity proteins. We have briefly discussed the possibility of engineering both enzyme specificity and substrate sequence for improved catalytic efficiency as well as mapping epitopes using short peptide libraries. In conclusion, bacterial cell surface display is a versatile and robust methodology that has been used successfully to develop novel high‐affinity proteins, and several of these have been evaluated preclinically for various disease indications, including cancer, neurodegenerative diseases, and inflammation, both as therapeutic drugs and diagnostic tracers with promising results.

## AUTHOR CONTRIBUTIONS

**Charles Dahlsson Leitao:** Writing – original draft; writing – review and editing. **Stefan Ståhl:** Writing – review and editing. **John Löfblom:** Writing – review and editing.

## FUNDING INFORMATION

This work was supported by grants from the Knut and Alice Wallenberg Foundation (grants KAW 2019.0341, KAW 2021.0197 and Wallenberg Center for Protein Research ‐ WCPR) SS; the Swedish Cancer Society (CAN23 2717 Pj: JL 20 1090 PjF; JL; 19 0101 Pj01H, SS 22 2023 Pj01H, SS); Hjärnfonden FO2022‐0253, FO2023‐0141, JL; the Swedish Research Council(2019–05115: JL); StratNeuro JL, and the Swedish Agency for Innovation VINNOVA (2019/00104 and CellNova center; 2017/02105 JL).

## CONFLICT OF INTEREST STATEMENT

S. S. is a minority shareholder in Affibody AB. C. D. L. and J. L. declare no conflict of interest.
